# Smoking in combination with antibodies to cyclic citrullinated peptides is associated with persistently high levels of survivin in early rheumatoid arthritis: a prospective cohort study

**DOI:** 10.1186/ar4438

**Published:** 2014-01-16

**Authors:** Björn Svensson, Ingiäld Hafström, Malin C Erlandsson, Kristina Forslind, Maria I Bokarewa

**Affiliations:** 1 Section of Rheumatology, Institute of Clinical Sciences, University Hospital, 22185 Lund, Sweden; 2 Department of Rheumatology, Karolinska Institute, Karolinska University Hospital Huddinge, 14159 Huddinge, Stockholm, Sweden; 3 Department of Rheumatology and Inflammation Research, Sahlgrenska University Hospital, University of Gothenburg, 40530 Gothenburg, Sweden; 4 Section of Rheumatology, Helsingborg Hospital, 25437 Helsingborg, Sweden

## Abstract

**Introduction:**

High levels of the oncoprotein survivin may be detected in the majority of patients with early rheumatoid arthritis (RA). Survivin is a sensitive predictor of joint damage and persistent disease activity. Survivin-positive patients are often poor responders to antirheumatic and biological treatment. The aim of this study was to investigate the reproducibility of survivin status and its significance for clinical and immunological assessment of RA patients.

**Methods:**

Survivin levels were measured in 339 patients from the Better Anti-Rheumatic FarmacOTherapy (BARFOT) cohort of early RA at baseline and after 24 months. The association of survivin status with joint damage (total Sharp-van der Heijde score), disease activity (Disease Activity Score based on evaluation of 28 joints (DAS28)), functional disability (Health Assessment Questionnaire (HAQ)), and pain perception (Visual Analogue Scale (VAS)) was calculated in the groups positive and negative for survivin on both occasions, and for the positive-negative and negative-positive groups.

**Results:**

In 268 patients (79%) the levels of survivin were similar at baseline and after 24 months, 15% converted from survivin-positive to survivin-negative, and 5% from survivin-negative to survivin-positive. A combination of smoking and antibodies against cyclic citrullinated peptides (aCCP) predicted persistently (baseline and 24 months) high levels of survivin (odds ratio 4.36 (95% CI: 2.64 to 7.20), *P* < 0.001), positive predictive value 0.66 and specificity 0.83). The independent nature of survivin and aCCP was demonstrated by statistical and laboratory analysis. Survivin positivity on both test occasions was associated with the progression of joint damage, significantly higher DAS28 and lower rate of remission at 24 and 60 months compared to negative-negative patients. Survivin status was less associated with changes in HAQ and VAS.

**Conclusions:**

Survivin is a relevant and reproducible marker of severe RA. Persistently high levels of survivin were associated with smoking and the presence of aCCP and/or RF antibodies and predicted persistent disease activity and joint damage.

## Introduction

The course of rheumatoid arthritis (RA) may vary considerably, from early and long-lasting remission to persistent disabling joint damage [1,2]. Early efficient antirheumatic treatment and tight follow-up have been a successful strategy to postpone disability. Identifying the patients with urgent need for antirheumatic treatment continues to be challenging. Reliable predictors of the disease progress and outcome assist clinicians to make the decision to choose and start antirheumatic treatment. Several models designed to predict the course of RA have recently been proposed. These models include ultrasound [3] and radiometric [4] evaluation of joints and a combination of genetic and environmental variables [5]. Serological markers remain a sensitive and reliable way of quick screening of broad patient cohorts. Antibodies against cyclic citrullinated peptides (aCCPs) and antibodies against the Fc domain of immunoglobulins (rheumatoid factor (RF)) are currently used to identify patients with a high probability of developing aggressive and therapy-resistant RA [6-9]. However, their predictive ability in individual patients is hampered by limited specificity.

We have recently found that RA patients with high levels of oncoprotein survivin in serum and synovial fluid have persistent joint inflammation and damage [10]. The proportion of survivin-positive patients may vary between 60% in patients with early RA [11] and 28% in patients with established RA undergoing treatment [10]. Survivin is a multifunctional protein that regulates cell proliferation and supports cell cycle progression and resistance to apoptosis [12]. In the rheumatoid synovia, survivin has been found at the sites of accumulation of macrophages and memory T cells in the inflamed tissues and in association with the transition to an invasive phenotype of synovial fibroblasts [13-15]. As a consequence of these cellular events, survivin may help to identify patients with early RA at risk of developing joint damage [11]. The results obtained in an independent cohort of RA patients showed that survivin was associated with a therapy-resistant course of arthritis, poor response to biological treatment [16] and a low remission rate [11]. Survivin is frequently found in conjunction with aCCPs and RF, and the combination of survivin with these autoantibodies enhances the predictive power of survivin.

The present study addresses the question of reproducibility of survivin status in consecutive tests of RA patients and the value of repeated survivin testing for prediction of joint damage, persistence of disease activity, functional disability and pain.

## Methods

### Patient cohort

A total of 339 patients with early RA (66% women, mean age 56.5 years, 55% RF-positive and 49% aCCP-positive) and a disease duration of 1 year or less were consecutively included into a multicentre observational study (the Better Anti-Rheumatic FarmacOTherapy (BARFOT) project) [7] between September 1993 and December 1999. All the patients fulfilled the classification criteria for RA established by the American Rheumatism Association [17]. No patient had received prior disease-modifying antirheumatic drug (DMARD) or prednisolone treatment for their RA. At the time of inclusion, each patient’s smoking history was recorded and defined as current, previous or never-smoker. Clinical and laboratory assessments of the patients were performed at the time of inclusion into the study (baseline) and thereafter at 6, 12, 24 and 60 months of follow-up. Radiography was performed at the time of inclusion and thereafter at 12, 24 and 60 months. The baseline data are presented in Table 1. All patients gave their informed consent and the ethics committees at Karolinska University Hospital, Lund University Hospital, Sahlgrenska University Hospital in Gothenburg and Linköping University Hospital approved the study. Disease activity was measured on the basis of the Disease Activity Score based on evaluation of 28 joints (DAS28) [18]. Remission was defined as DAS28 <2.6 (European League Against Rheumatism criteria) [19]. Functional disability was assessed using a Swedish version of the Stanford Health Assessment Questionnaire (HAQ) [20]. Pain perception was assessed on a Visual Analogue Scale (VAS) with a range from 0 to 100 mm.

**Table 1:** **Clinical and demographic baseline characteristics of rheumatoid arthritis patients**^**a**^

**Patient demographics (*N*** **= 339)**	**Level and frequency**
Age, years	56.5 ± 16
Women	224 (66%)
Disease duration, months	6.2 ± 3.3
RF^+^	184 (55%)
aCCP^+^	169 (49%)
DAS28	5.6 ± 1.26
VAS pain, mm	45 ± 24
HAQ	1.00 ± 0.66
SHS, median (IQR)	1.7 (0 to 5)
Smoking habits	
Never	166 (49%)
Previous	89 (26%)
Current	84 (25%)
Treatment at start	
MTX	131 (39%)
SLZ	112 (33%)
Combination of DMARDs	3 (1%)
No DMARDs	51 (15%)
Prednisolone	210 (62%)

^a^aCCP^+^, positive for antibodies against cyclic citrullinated peptides; DAS28, Disease Activity Score based on evaluation of 28 joints; DMARD, disease-modifying antirheumatic drug; HAQ, Health Assessment Questionnaire; MTX, methotrexate; RF^+^, rheumatoid factor–positive; SHS, Sharp-van der Heijde score; SLZ, sulphasalazine; VAS, Visual Analogue Scale. The values are mean ± SD or *n* (%) unless otherwise stated.

### Laboratory analyses

Serum samples were collected at the time of inclusion into the study and after 24 months and stored at -70°C until further analyses. The levels of survivin were measured by performing a sandwich enzyme-linked immunosorbent assay (ELISA) (DYC647; R&D Systems, Abingdon, UK). Values of circulating survivin above 300 pg/ml, corresponding to 3 standard deviations over the mean level of a healthy control group, were defined as positive [10]. RF was analysed using the SERODIA-RA rheumatoid factor agglutination test (Fujirebio, Tokyo, Japan). Positive RF was defined as a titre of >20 IU/ml. aCCPs were detected by ELISA (anti-CCP2 antibody kit; Euro Diagnostica AB, Malmö, Sweden), positive aCCP was defined as >25 IU/ml.

### Technical evaluation of survivin by ELISA for potential interference of autoantibodies

Twenty randomly chosen serum samples with known levels of survivin, RF and aCCP were used to prepare a survivin-positive pool (pool A), a RF-positive pool (pool B), a RF- and aCCP-positive pool (pool C) and a negative pool (pool D) (Table 2). The following experimental procedures were carried out: serial dilution of pool A to test recovery of survivin levels with sample dilution; a mixture procedure whereby pool A (survivin-positive) was diluted with pool B (RF-positive); and a spike recovery procedure whereby pool D (negative), pool B (RF-positive) and pool C (RF- and aCCP-positive) were spiked with recombinant survivin (10 ng/ml, 5 ng/ml, 2.5 ng/ml, 1.25 ng/ml and 0 ng/ml).

**Table 2:** **Individual measurements of survivin, rheumatoid factor and** a**ntibodies against cyclic citrullinated peptides in the randomly selected serum samples used for evaluation of the serum survivin enzyme-linked immunosorbent assay**^**a**^

**Serum pools, sample no**			**RF (IU/ml)**		**CCP (IU/ml)**		**Survivin (ng/ml)**	
				**Mean**		**Mean**		**Mean**
A	RF^-^	1	0	0	0	0	7.45	20.3
	aCCP^-^	2	0		0		40.7	
	Surv^+^	3	0		0		35.4	
		4	0		0		8.07	
		5	0		0		9.95	
B	RF^+^	6	160	120	0	0	0	0
	aCCP^-^	7	83		0		0	
	Surv^-^	8	240		0		0	
		9	65		0		0	
		10	50		0		0	
C	RF^+^	11	160	202	480	534	0	0
	aCCP^+^	12	140		590		0	
	Surv^-^	13	220		51		0	
		14	390		48		0	
		15	100		1500		0	
D	RF^-^	16	0	0	0	0	0	0
	aCCP^-^	17	0		0		0	
	Surv^-^	18	0		0		0	
		19	0		0		0	
		20	0		0		0	

^a^aCCP, antibodies against cyclic citrullinated peptide; RF, rheumatoid factor; Surv, survivin.

**Figure 1:**
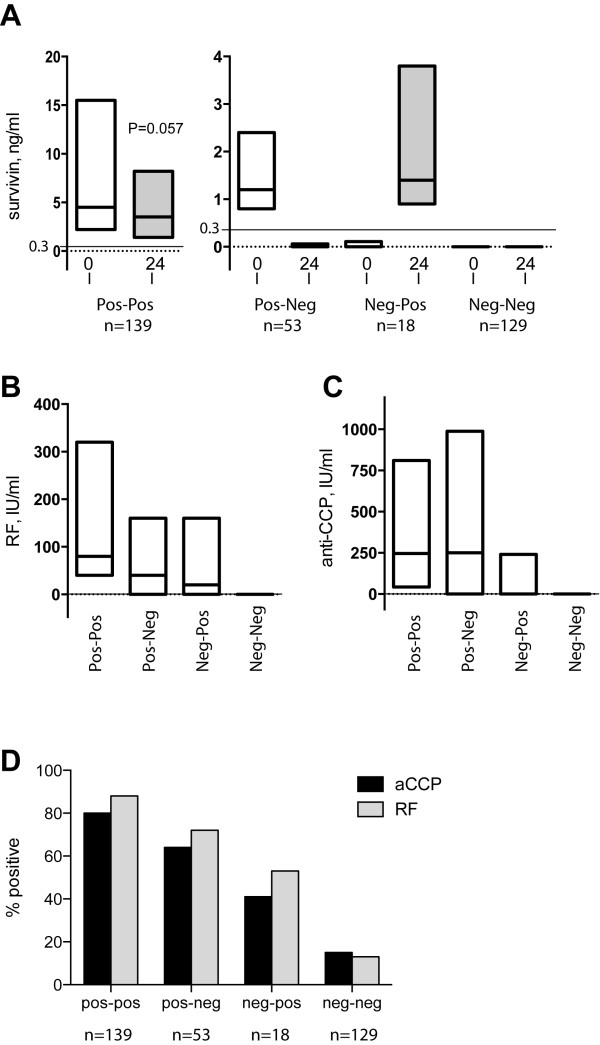
**Absolute serum levels of survivin, rheumatoid factor and antibodies against cyclic citrullinated peptide in the rheumatoid arthritis patients.** The levels of survivin were measured in 339 patients from the Better Anti-Rheumatic FarmacOTherapy (BARFOT) cohort of early rheumatoid arthritis at baseline and after 24 months. The patients were divided into four groups based on their survivin status at those two occasions indicated as positive-positive (*n* = 139), positive–negative (*n* = 53), negative–positive (*n* = 18) and negative-negative (*n* = 129). **(A)** The absolute levels of survivin are shown at baseline and 24 months. The concentration >0.3 ng/ml indicates high survivin levels (shown as a dashed line). The absolute levels of rheumatoid factor (RF) (IU/ml) **(B)** and cyclic citrullinated peptide (CCP) (IU/ml) **(C)** are shown at baseline. Data are shown as IQRs with the medians indicated by lines. **(D)** The frequency of autoantibodies (RF and antibodies against CCP (aCCP)) within each patient group is shown (%). The Wilcoxon signed-rank test was used for comparison of survivin levels at baseline and 24 months.

### Radiographic measurements

Posteroanterior radiographs of the hands and feet were obtained at the time of study entry from all 339 patients, at 24 months from 337 patients and at 60 months from 296 patients. Radiographic joint damage was assessed on the basis of the Sharp-van der Heijde score (SHS) [21]. The films were read by one of two experienced readers as previously described [11]. Double-readings of a fraction of films showed good agreement between the two readers. For 49 such double-readings of baseline and 5-year films, the smallest detectable change (SDC) could be calculated by defining radiological progression as a >5.4 change in the total SHS score over the course of 5 years. Radiographic progression was defined as a >5.4 change in total SHS over the course of 60 months, the SDC.

### Statistical analysis

Statistical analyses were performed using SPSS version 21.0 statistical software (SPSS, Chicago, IL, USA). To test the differences between groups, the Mann–Whitney *U* test, the Kruskal–Wallis test and the Wilcoxon signed-rank test for paired analysis were used for continuous variables, and the χ^2^ test was used for proportions. When an overall significance was obtained by the Kruskal–Wallis test, pairwise *post hoc* analyses were calculated using SPSS software (nonparametric tests of two or more independent samples). Spearman’s rank correlation coefficient was used to assess the relationships between two continuous variables. All significance tests were two-tailed and conducted at the 0.05 level of significance. Univariate analyses of the association of survivin and other baseline clinical and demographic variables with radiological and clinical outcomes were performed. The predictive performance of survivin was analysed by 2 × 2 tables and odds ratios (ORs), sensitivity, specificity, positive predictive value (PPV) and negative predictive value were calculated.

## Results

### Variability of survivin status over 24 months

The levels of survivin were measured in the paired serum samples of 339 patients at the first visit to a rheumatologist (baseline) and 24 month later. The absolute levels of survivin in the total material changed from 0.95 ng/ml (0 to 4.3) at baseline to 0.22 ng/ml (0 to 2.5) at 24 months (median (IQR); *P* < 0.001). Survivin-positive samples (>0.3 ng/ml) comprised 56.6% (192 of 339 patients) of the baseline samples and 46.3% (157 of 339 patients) of the samples taken 24 months later. Survivin status at baseline was similar to that at baseline in 79% of samples taken at 24 months, referred to below as positive-positive and negative-negative groups, respectively. The absolute values of the positive-positive group tended to be lower after 24 months (*P* = 0.057) (Figure 1A). A switch from survivin-positive to survivin-negative occurred in 53 samples (15.6%), defined as positive–negative, and 18 (5.3%) of the samples negative for survivin at baseline converted to positive 24 months later. The latter samples are referred to below as negative–positive.

The changes in survivin status occurred equally often in the patients treated with methotrexate or sulphasalazine as in those not treated with DMARDs. The change in survivin status occurred independently of treatment with low-dose oral prednisolone. Thus, we observed no relationship between survivin status and treatment started at baseline. At 24 months, the positive-positive patients were significantly more often on DMARD treatment including combination of two or more DMARDs compared to the negative-negative patients (81.3% vs 50.4%, *p* = 0.001).

### Predictive role of autoantibodies and smoking on survivin status

Positivity for survivin at baseline was associated with cigarette smoking status in 71% of current smokers, 56% of previous smokers and 49% of never smokers (overall *P* = 0.004). The OR for survivin positivity at baseline was significantly higher in the patients who had ever smoked compared to nonsmokers (OR 1.79 (95% CI 1.16 to 2.76); *P* = 0.009). A change in survivin status occurred equally as often in smokers as in nonsmokers.

The ability of smoking, aCCP and RF to predict the persistence of survivin positivity was assessed. It was found that survivin positivity persisted more frequently in patients who had ever smoked than in nonsmokers (OR 1.81 (95% CI 1.17 to 2.81); *P* = 0.008). Furthermore, survivin positivity on both test occasions was also more common in the patients who were aCCP-positive and/or RF-positive compared with the rest of the patients (OR 15.56 (95% CI 8.06 to 30.02); *P* < 0.001). Similarly, survivin persistence was more common in the aCCP-positive (OR 8.89 (95% CI 5.32 to 14.87); *P* < 0.001) and RF-positive patients (OR 14.67 (95% CI 8.14 to 26.44); *P* < 0.001). Patients with a history of smoking combined with the presence of aCCP were more often survivin-positive on both test occasions than the rest of the patients (OR 4.36 (95% CI 2.64 to 7.20); *P* < 0.001). This was also the case for patients with a history of smoking combined with aCCP and/or RF positivity (OR 4.60 (95% CI 2.85 to 7.45); *P* < 0.001). These data indicate that smoking status and the presence of aCCP and/or RF have an additive value for predicting persistently high levels of survivin in RA patients.

### Accuracy of ELISA for survivin in presence of autoantibodies

The presence of autoantibodies (RF and aCCP) was detected in a significant number of survivin-positive samples (Figure 1D) on the basis of absolute values (in IU/ml) of RF (Figure 1B) and aCCP (Figure 1C), and correlation between these biological markers was noted (survivin/aCCP: Spearman’s ρ = 0.59, survivin/RF: Spearman’s ρ = 0.72, aCCP/RF: Spearman’s ρ = 0.65; *P* < 0.001 for each pair). In a logistic regression model in our previous article [11], survivin and aCCP, but not RF, were independent predictors of radiological progression, whereas RF did not. When we repeated this calculation in the present, somewhat smaller cohort, we obtained similar results (survivin: OR 2.28 (95% CI 0.99 to 5.23), *P* < 0.052; aCCP: OR 5.72 (95% CI 2.36 to 13.86), *P* < 0.001; RF: OR 0.69 (95% CI 0.26 to 1.82), *P* = 0.45).

**Figure 2:**
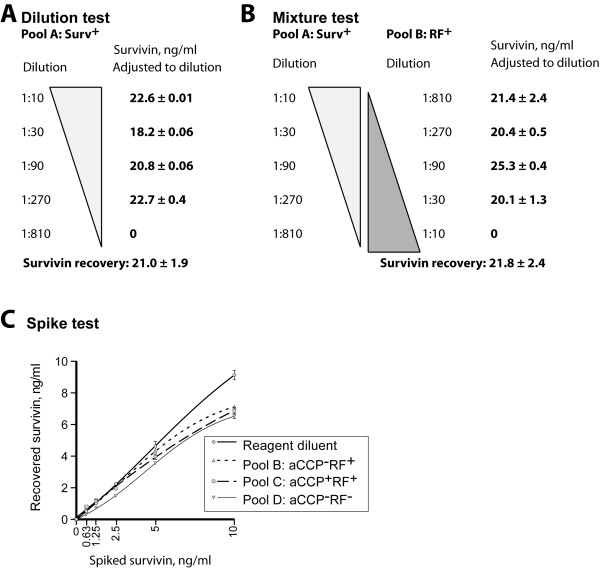
**Evaluation of potential interference of autoantibodies with measurements of survivin by ELISA. (A)** Twenty randomly chosen serum samples with known levels of survivin (Surv), rheumatoid factor (RF) and autoantibodies to cyclic citrullinated peptides (aCCPs) were used to prepare a survivin-positive pool (pool A), a RF-positive pool (pool B), a RF- and aCCP-positive pool (pool C) and a RF- and aCCP-negative pool (pool D). Serial dilution of pool A showed 98% recovery of survivin levels with sample dilution. **(B)** Pool A (survivin-positive) was diluted with pool B (RF-positive). The measurements of survivin were not affected by these increasing concentrations of RF. **(C)** A spike recovery procedure whereby pool D (RF- and aCCP-negative), pool B (RF–positive and aCCP-negative) and pool C (RF- and aCCP-positive) were spiked with recombinant survivin. The measurements of survivin showed 85% to 100% recovery, which was similar for all tested pools.

Potential interference of autoantibodies in the measurement of survivin levels was addressed. First, we studied the recovery of survivin levels using serial dilution of pooled samples with known concentrations of survivin (pool A). The recovery of survivin measurements was 98% of the expected concentration (Figure 2A). In the second step, a mixing procedure was performed whereby the survivin-positive pool was diluted with RF-positive pool, which produced increasing concentrations of RF with each dilution. The resulting concentrations did not differ from the parallel samples without RF (Figure 2B), and the recovery of survivin measurements was 98%. Third, a spike recovery procedure was used whereby pool B (RF-positive) and pool C (RF- and aCCP-positive) and pool D (RF- and aCCP-negative) were spiked with predetermined concentrations of recombinant survivin between 0 and 10 ng/ml. Levels of survivin measured in each pooled sample showed the recovery of 85% to 100% in the different concentrations tested (Figure 2D). Taken together, the experimental procedures proved the accuracy of survivin measurements in samples containing autoantibodies (RF and/or aCCP).

### Radiographically detected joint damage and progression

**Figure 3:**
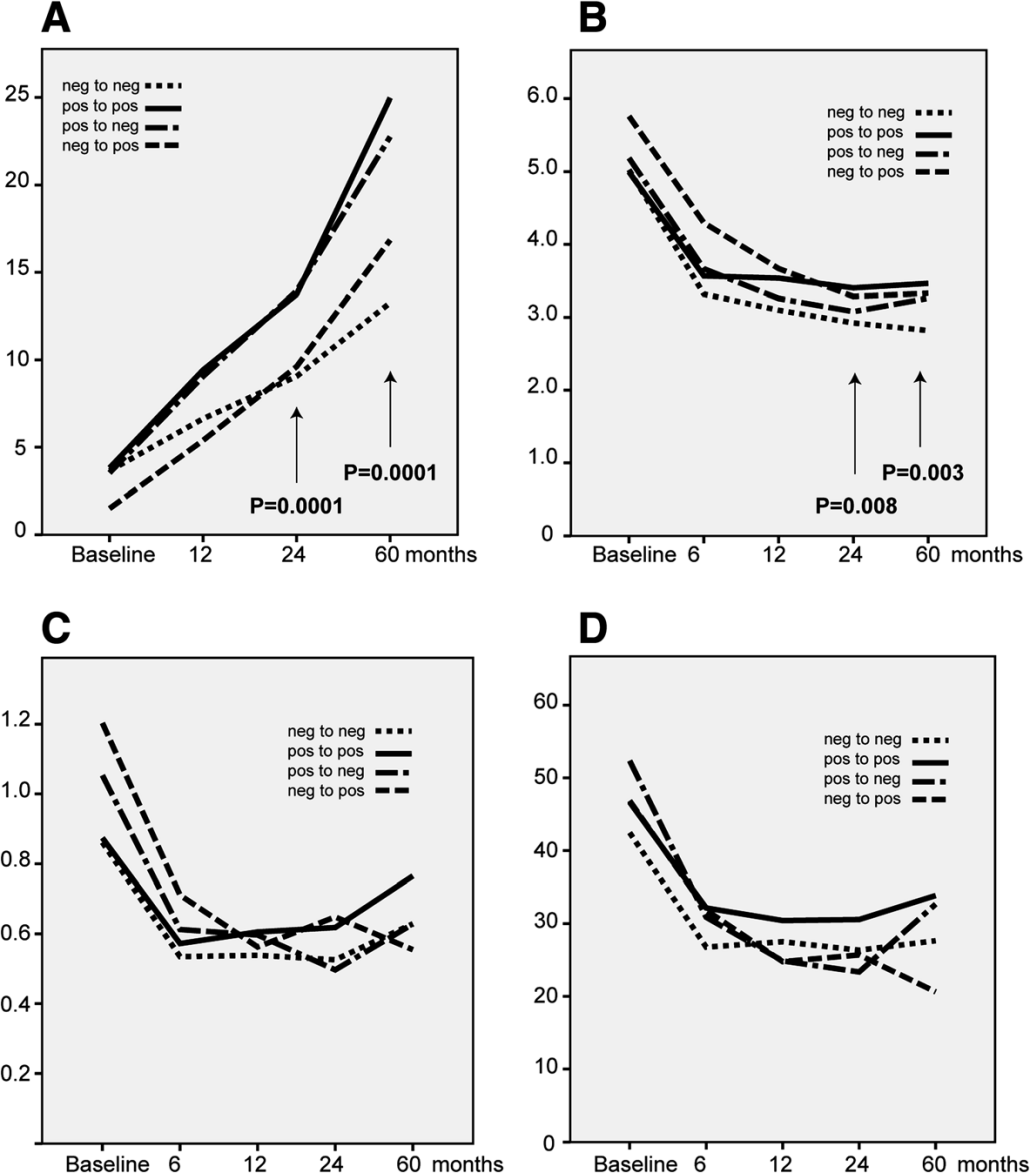
**Survivin positivity is associated with persistent disease activity and progressive joint damage in rheumatoid arthritis patients.** The development of radiographically detected joint damage (assessed by Sharp-van der Heijde score SHS)) **(A)**, Disease Activity Score based on evaluation of 28 joints (DAS28) **(B)**, functional disability (Stanford Health Assessment Questionnaire (HAQ)) **(C)** and pain perception (pain scored on Stanford Visual Analogue Scale (VAS)) **(D)** are presented in the positive and negative groups for survivin on both occasions (positive-positive (*n* = 139) and negative-negative (*n* = 129)) and for the positive–negative group (*n* = 53) and negative–positive group (*n* = 18). There was a significant overall difference in SHS between groups at 2 and 5 years (*P* = 0.001 for both time points). Similarly, overall significant differences in DAS28 scores between groups were observed at 2 and 5 years (*P* = 0.008 and 0.003, respectively).

Our previous study showed that survivin positivity at baseline strongly predicted the development and progression of joint damage [11]. In our present study, we addressed the question whether a change of survivin status affects the development of radiographic joint damage. Figure 3A shows the median increase in total SHS over time in the four survivin groups. The overall and *post hoc* analysis between the individual groups revealed that the positive-positive group had a greater increase in SHS than the negative-negative group at 24 and 60 months (both, *P* = 0.0001). The *post hoc* pairwise analyses showed significant differences between the positive-positive and negative-negative groups (*P* = 0.001 at 2 years and *P* < 0.001 at 5 years) and the positive-negative and negative-negative groups (*P* = 0.002 at 2 years and *P* = 0.001 at 5 years). Survivin positivity both at baseline and after 24 months predicted radiographically detected progression at 60 months (OR 2.9 (95% CI 1.7 to 5.1), *P* = 0.001; PPV 0.72 and specificity 0.75).

### Clinical remission and functional disability

The change in DAS28 scores in the four analysed groups during 60 months of follow-up are presented in Figure 3B. Because the overall significant differences between survivin groups, the *post hoc* analyses between individual groups were performed. Overall significant differences in DAS28 scores between groups was observed at 2 and 5 years (*P* = 0.008 and 0.003, respectively). *Post hoc* comparisons displayed significant differences between the negative-negative and positive-positive groups at 2 years (*P* = 0.04) and at 5 years (*P* = 0.004).

A univariate analysis of the ability of survivin status to predict remission at 60 months showed that only survivin negativity both at baseline and at 24 months predicted remission after 60 months (OR 2.14 (95% CI 1.3 to 3.5), *P* = 0.002; PPV 0.50 and specificity 0.69). After adjusting for RF, the survivin negativity on both occasions still predicted remission at 60 months (OR 1.99 (95% CI 1.02 to 3.87); *P* = 0.043). The probability of achieving remission within 60 months was low in patients positive for survivin on any test occasion. Figures 3C and 3D show the development of pain perception (VAS-rated pain) and functional disability (based on HAQ) in the four survivin groups. No statistically significant effect of survivin status on these variables was observed. However, the patients who were positive for survivin on both test occasions tended to develop the highest HAQ scores (Figure 3C) and had persistently high VAS-rated pain (Figure 3D) during the follow-up period.

## Discussion

In this prospective study, we show that the presence of survivin in patients with early-onset RA is a consistent and repeatable finding. The cellular origin and processes, which lead to extracellular accumulation of survivin in the blood and synovial fluid of RA patients remains elusive. The cells of nontumoural origin, thymocytes and CD34^+^ bone marrow stem cells comprise the major natural pool of survivin. Survivin is preferentially expressed during mitotic phases of the cell cycle, and its expression is downregulated at interphase [22]. Deregulation of the *survivin* gene in transformed cells results in its overexpression at all phases of the cell cycle. Survivin is also overexpressed in different types of tumours [23]. In RA patients, survivin is found in the proliferating synovial fibroblasts of the lining layer and in the macrophages and T cells of the inflamed synovial tissue [13-15]. Immunomediated destruction of the transformed cells overexpressing survivin by cytotoxic T cells has been described in patients with multiple myeloma [24] and neuroblastoma [25], which may provide a plausible explanation for the extracellular appearance of survivin. Exosomal release of survivin to the extracellular space has been described previously [26]. The reproducibility of survivin status in the consecutive tests in RA patients in the present study suggests that the release of survivin accompanies cellular events and is associated with clinical disease activity and radiographically detected joint damage.

The switch of survivin status from positive to negative that we observed in some of RA patients 24 months after inclusion into the study could reflect a cessation of cytolysis due to a regression of inflammation and a reversion of the pathological process. The initial observation of an association between survivin and progressive joint damage in early RA is supported by the data in the present study, in which the SHS continued to increase in patients positive for survivin at baseline, regardless of their later survivin status. In addition, those patients who converted to survivin positivity after 24 months tended to display accelerated joint damage later.

The study design permitted only limited analyses of survivin status with respect to the effect of methotrexate, sulphasalazine and low-dose prednisolone treatment. We observed no association between survivin status and DMARD treatment. No coherence was found between any of these DMARDs and a survivin switch in the available patient cohort. The obvious limitation of the study is the lack of patients treated with biologic drugs, which are known to efficiently stop disease activity and joint damage in RA patients resistant to methotrexate treatment [27]. A switch from survivin-positive to survivin-negative status is expected, followed by arrest of radiographically observed progression.

Intriguingly, survivin is also associated with the presence of RF and aCCP, the markers of deep changes in self-recognition in RA patients. The presence of survivin-specific immune reactions would be expected, owing to intense production of the antisurvivin antibodies measured in RA patients [10], and may be similar to the mechanisms triggering the production of autoantibodies. The high correlation observed between the presence of autoantibodies (RF and/or aCCP) and survivin obligated us to study the accuracy of survivin measurements in the samples containing RF and/or aCCP. Three steps of technical evaluation, including dilution-related recovery and spike recovery procedures, allowed us to conclude that the presence of autoantibodies is unlikely to affect the measurement of survivin in the experimental setting we used. Additionally, the statistical evaluation proved the independent nature of these parameters for the prediction of radiologically observed progression of joint damage.

In this study, survivin was associated with the presence of aCCP and with smoking history at baseline. This association draws attention to a known predictive value of smoking for the development of RA ahead of its clinical symptoms [28,29]. Whether high levels of survivin may be considered a consequence of smoking is not clear. We found a recent report of measurements of serum survivin in patients with non-small cell lung cancer, among whom 92% were smokers [30]. The study failed to identify differences in survivin levels between the lung cancer patients and nonsmoker controls. However, more studies of this interesting issue must be awaited. Interestingly, the presence of aCCP and smoking was predictive for persistent survivin positivity. aCCP has been found to be an independent predictor of radiographically visualised joint damage [7,31] and the efficacy of DMARD treatment [32] in patients with established RA. We have previously reported that a combination of survivin with aCCP and/or RF enhances the predictive power for the development of joint damage [11]. A multiple logistic regression analysis revealed survivin as a factor independent of aCCP for the prediction of radiographically observed joint damage. The evaluation of the predictive role of survivin for the development of RA would be a natural next step in understanding the role of survivin in the pathogenesis of the disease.

## Conclusion

Survivin is a relevant and reproducible marker of severe RA. Survivin positivity on both test occasions was associated with smoking and the presence of aCCP and/or RF antibodies, as well as with persistent disease activity and joint damage. Survivin may be included in predictive models for improved treatment decisions for patients with early RA.

## Abbreviations

aCCP: antibodies against cyclic citrullinated peptide; BARFOT: Better Anti-Rheumatic FarmacOTherapy; CI: confidence interval; CRP: C-reactive protein; DAS28: Disease Activity Score based on evaluation of 28 joints; DMARD: disease-modifying antirheumatic drug; ELISA: enzyme-linked immunosorbent assay; ESR: erythrocyte sedimentation rate; HAQ: Health Assessment Questionnaire; IQR: interquartile range; OR: Odds ratio; PPV: Positive predictive value; RA: rheumatoid arthritis; RF: rheumatoid factor; SD: standard deviation; SHS: Sharp-van der Heijde scoring method for evaluation of radiographic joint damage; VAS: Visual Analogue Scale

## Competing interests

The authors declare that they have no competing interests.

## Authors’ contributions

All co authors fulfil the criteria of authorship. MB initiated the study. BS, KF and IH performed the clinical examination of the patients. ME and MB were responsible for laboratory analysis. KF read the radiographs. BS, ME and MB carried out statistical evaluation of the data. BS, ME, MB, IH and KF prepared the manuscript. All authors read and approved the final version of the manuscript.
